# A plant-based diet in overweight individuals in a 16-week randomized clinical trial: metabolic benefits of plant protein

**DOI:** 10.1038/s41387-018-0067-4

**Published:** 2018-11-02

**Authors:** Hana Kahleova, Rebecca Fleeman, Adela Hlozkova, Richard Holubkov, Neal D. Barnard

**Affiliations:** 1 Physicians Committee for Responsible Medicine, 5100 Wisconsin Ave, N.W. Ste.400, Washington, DC 20016 USA; 2 School of Medicine, University of Utah, Salt Lake City, UT 84132 USA; 3 Adjunct Faculty, George Washington University School of Medicine and Health Sciences, Washington, DC 20016 USA

## Abstract

**Background and objectives:**

A plant-based diet is an effective strategy in the treatment of obesity. In this 16-week randomized clinical trial, we tested the effect of a plant-based diet on body composition and insulin resistance. As a part of this trial, we investigated the role of plant protein on these outcomes.

**Subjects and methods:**

Overweight participants (*n* = 75) were randomized to follow a plant-based (*n* = 38) or a control diet (*n* = 37). Dual X-ray Absorptiometry assessed body composition, Homeostasis Model Assessment (HOMA-IR) assessed insulin resistance, and a linear regression model was used to test the relationship between protein intake, body composition, and insulin resistance.

**Results:**

The plant-based vegan diet proved to be superior to the control diet in improving body weight, fat mass, and insulin resistance markers. Only the vegan group showed significant reductions in body weight (treatment effect −6.5 [95% CI −8.9 to −4.1] kg; Gxt, *p* < 0.001), fat mass (treatment effect −4.3 [95% CI −5.4 to −3.2] kg; Gxt, *p* < 0.001), and HOMA-IR (treatment effect −1.0 [95% CI −1.2 to −0.8]; Gxt, *p* = 0.004). The decrease in fat mass was associated with an increased intake of plant protein and decreased intake of animal protein (*r* = -0.30, *p* = 0.011; and *r* = +0.39, *p* = 0.001, respectively). In particular, decreased % leucine intake was associated with a decrease in fat mass (*r* = +0.40; *p* < 0.001), in both unadjusted and adjusted models for changes in BMI and energy intake. In addition, decreased % histidine intake was associated with a decrease in insulin resistance (*r* = +0.38; *p* = 0.003), also independent of changes in BMI and energy intake.

**Conclusions:**

These findings provide evidence that plant protein, as a part of a plant-based diet, and the resulting limitation of leucine and histidine intake are associated with improvements in body composition and reductions in both body weight and insulin resistance.

## Introduction

Suboptimal nutrition is a major cause of obesity, chronic disease, and premature death across the nation and worldwide^1,2^. Certain dietary habits, such as high intakes of sodium and processed meat products and low intakes of fruits and vegetables, are associated with 45.5% of cardio-metabolic deaths in the United States^3^. Fortunately, research has shown a plant-based vegan diet to be beneficial in improving nutrient intake^4^, decreasing all-cause mortality, and decreasing risk of obesity, type 2 diabetes, and coronary heart disease^5^.

A plant-based vegan diet excludes all animal products and is centered around grains, legumes, vegetables, and fruits. While adequate in macro and micronutrients^6^, people sometimes question the ability to reach protein requirements on a plant-based vegan diet. A sufficient protein intake is necessary to supply nitrogen and amino acids to our cells to ensure the growth and maintenance of the protein pool in our bodies^7^. However, a diet based entirely on plants provides all essential amino acids and an adequate quantity of overall protein, even without the use of special food combinations^6^. Further, the consumption of exclusively plant proteins has been associated with reduction of the concentrations of blood lipids^8–11^, obesity^12^, and cardiovascular disease^13–15^.

The specific composition of dietary protein has been shown to influence the balance of glucagon and insulin activity^14^, which may play a role in body composition and insulin resistance^12^. A high intake of branched chain amino acids (leucine, isoleucine, and valine) can increase insulin resistance^16^. In addition, dietary restriction of sulfur containing amino acids (methionine and cysteine), is associated with a reduction in body weight, adiposity and metabolic changes in both adipose and liver tissues, which enhance insulin sensitivity and energy expenditure^17^. Plant protein low in sulfur also reduces blood lipids, homocysteine, and blood pressure^18,19^. Furthermore, low protein diets are also associated with increased life span, especially if the consumed protein is plant derived^20^.

In this secondary analysis of data from a 16-week randomized clinical trial^21^, we explore the effects of plant protein, as part of a plant-based diet, on weight control, body composition, and insulin resistance in overweight individuals.

## Materials/subjects and methods

### Study design

The study was conducted between October 2016 and June 2017, using a single-center, randomized, open parallel design. Otherwise healthy overweight or obese adult men and women, with a body-mass index between 28 and 40 kg/m^2^, were enrolled. Exclusion criteria were history of diabetes, smoking, alcohol or drug abuse, pregnancy or lactation, and current use of a vegan diet. The study protocol was approved by the Chesapeake Institutional Review Board on October 12, 2016. All participants signed a written informed consent. Registration on ClinicalTrials.gov was initiated on October 20, 2016 (Identifier: NCT02939638).

### Randomization and study groups

Participants were randomly assigned in a 1:1 ratio to a vegan or a control group based on a computer-generated randomization protocol. The randomization protocol was not be accessible beforehand. The particpants were not blinded to their group assignment. They were examined at baseline and 16 weeks. The vegan group was asked to follow a low-fat vegan diet consisting of vegetables, grains, legumes, and fruits. They were instructed to avoid animal products and added oils. Daily fat intake was 20–30 g. No meals were provided. Participants in the control group were asked to maintain their current diets, which included animal products, for the duration of the study. Laboratory measurements and statistical analyses were made by staff members blind to group assignment.

### Dietary intake and physical activity

To monitor adherence, a 3-day dietary record was completed by each participant at baseline and 16 weeks. Dietary intake data were collected and analyzed by a registered dietician, using Nutrition Data System for Research version 2016, developed by the Nutrition Coordinating Center, University of Minnesota, Minneapolis, MN^22^. The study participants were instructed not to change their physical activity, and to continue their chronic medications, except as modified by their personal physicians. Physical activity was assessed by the International Physical Activity Questionnaire^23^.

### Outcomes

All measurements were performed at baseline and 16 weeks on an outpatient basis, after a 10–12 h overnight water-only fast. Height was measured using a standiometer. Weight was measured using a periodically calibrated scale accurate to 0.1 kg. Body composition was measured using a DXA scan (iDXA; GE Healthcare, Chicago, IL, USA). Insulin resistance was evaluated using HOMA-IR (Homeostasis Model Assessment)^24^. Self-reported dietary intake of animal and plant protein was analyzed. Amino acid intakes were assessed and used as predictors of changes in body composition and insulin resistance.

### Statistical analysis

We based a calculation of the sample size on an alpha of 0.05 and 0.80 beta to detect between-group differences in outome variables: a clinically relevant 10% difference in insulin resitance (HOMA IR) and a 5% difference in BMI. We required 54 participants to complete the intervention they were randomised to. The intention-to-treat analysis included all participants. A repeated measure ANOVA model, that included factors group, subject, and time, was used to test the between-group differences throughout the 16 week study. Interaction between group and time (Gxt) was calculated for each variable. We tested the data for normal distribution. Within each diet group, paired comparison *t*-tests were calculated to test whether the change from baseline to 16 weeks was significantly different from zero. Pearson correlations were calculated for the relationship between changes in reported protein and amino acid intake on one side, and body composition and insulin resistance on the other. Values were first unadjusted and then adjusted for changes in BMI and energy intake. Regression analyses assessed the effect size of changes in animal and plant protein and of changes in amino acid intake on body composition and insulin resistance.

## Results

### Characteristics of the participants

The total randomized sample size was 75 participants, 96% (*n* = 72) of whom completed the study (see Supplemental Fig. 1). The mean age of participants was 53.2 ± 12.6 years and 89% (*n* = 67) of participants were women. Additional baseline characteristics can be found in  Table 1.

**Table 1:** Baseline characteristics of study population

Characteristic	*n* = 75
Age (years)	53.2 ± 12.6
Sex (number, %)	
Male	8 (11%)
Female	67 (89%)
Race, (number, %)	
White	34 (45%)
Black	34 (45%)
Asian, Pacific Islander	4 (5%)
American Indian, Eskimo, Aleut	2 (3%)
N/A—did not disclose	1 (1%)
Ethnicity, (number, %)	
Non-hispanic	64 (85%)
Hispanic	6 (8%)
N/A—did not disclose	5 (7%)
Education	
College	37 (49%)
Graduate degree	37 (49%)
NA	1 (1%)
Medications	
Lipid-lowering therapy (%)	9 (12%)
Antihypertensive therapy (%)	18 (24%)
Thyroid medications (%)	9 (12%)

### Physical activity and dietary intake

**Fig. 1:**
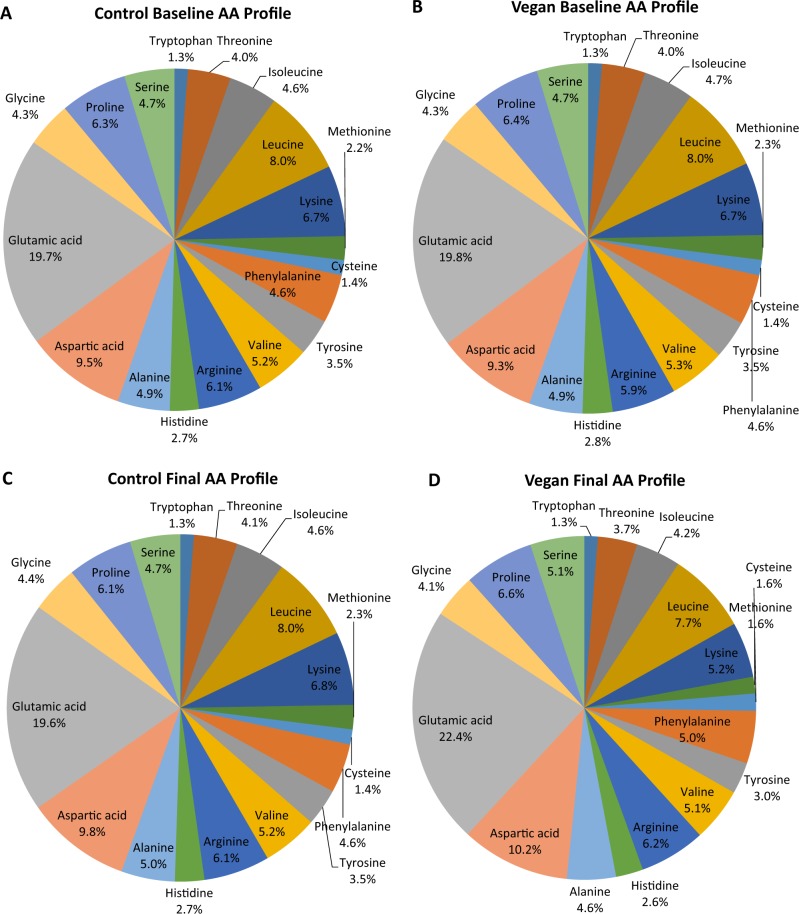
Changes in amino acid (AA) profiles between vegans and controls at baseline and 16 weeks. (A) Control Baseline, (B) Vegan Baseline (C) Control Final, (D) Vegan Final

Data on physical activity and dietary intake can be found in Table 2. Overall, physical activity remained consistent among both groups. Energy intake decreased across the study with no significant difference between groups. Total protein intake decreased in the vegan group but did not change in the control group (treatment effect, i.e. the treatment difference, −17.0 g; 95% CI −30.5 to −3.4). Controls did not change their ratio of sources of protein between baseline and 16 weeks. In contrast, the vegan group significantly increased plant protein intake (treatment effect, +19.2 g; 95% CI, +10.5 to +28.0) and decreased animal protein intake (treatment effect, −36.2 g; 95% CI, −48.4 to −24.0). The intake of branched chain amino acids (leucine, isoleucine, and valine) and histidine decreased significantly in the vegan group, but did not change in the control group. Detailed % intake of amino acids is shown in Fig. 1.

**Table 2:** Changes in dietary intake of amino acids (g) during the study.

	Control group		Vegan group		Treatment effect	*p*- value
Activity and diet	Baseline	Week 16	Baseline	Week 16		
Physical activity (METs)	2642 (1476–3809)	2575 (1169–3980)	2207 (1444–2969)	2490 (1586–3395)	+351 (−1143 to +1846)	0.46
Caloric intake (kcal.day^−1^)	1923 (1627–2219)	1582 (1368–1795)**	1851 (1695–2007)	1450 (1249–1652)***	−60 (−352 to+233)	0.69
Carbs (% of daily energy)	45.5 (42.6–48.4)	46.6 (42.9–50.4)	46.1 (43.5–48.8)	69.6 (67.3–71.8)***	+22.3 (+17.7 to +26.9)	<0.001
Fats (% of daily energy)	35.6 (32.3–38.9)	35.0 (31.5–38.4)	36.1 (34.0–38.1)	17.5 (15.5–19.4)***	−17.9 (−22.3 to −13.6)	<0.001
Proteins (% of daily energy)	16.00 (14.94–17.07)	16.99 (15.45–18.52)	16.77 (15.36–18.19)	12.26 (11.26–13.25)***	−5.50 (−7.90 to −3.11)	<0.001
Total protein (g/day)	75.4 (65.4–85.4)	66.8 (57.3–76.2)	76.5 (68.8–84.2)	50.8 (42.4–59.3)***	−17.0 (−30.5 to −3.4)	0.01
Animal protein (g)	45.16 (37.2–53.1)	39.68 (30.5–48.9)	45.01 (38.4–51.7)	3.34 (0.66–6.02)***	−36.2 (−48.4 to −24.0)	<0.001
Plant protein (g)	30.3 (25.8–34.7)	27.1 (22.8–31.4)	31.5 (26.7–36.2)	47.5 (39.7–55.3)***	+19.2 (+10.5 to +28.0)	<0.001
Cholesterol intake (mg.day^−1^)	290 (220–360)	212 (149–275)	264 (213–315)	6.5 (2.5-10.5)***	−180 (−278 to −82)	<0.001
Tryptophan (g)	0.92 (0.78–1.06)	0.80 (0.67–0.92)	0.93 (0.83–1.02)	0.61 (0.50–0.72)***	−0.19 (−0.38 to −0.01)	0.04
Threonine (g)	2.92 (2.51–3.33)	2.58 (2.19–2.97)	2.93 (2.62–3.25)	1.78 (1.47–2.09)***	−0.81 (−1.36 to −0.27)	0.004
Isoleucine (g)	3.32 (2.85–3.79)	2.94 (2.50–3.37)	3.41 (3.04–3.77)	2.03 (1.67–2.40)***	−0.99 (−1.62 to −0.35)	0.003
Leucine (g)	5.80 (5.01–6.59)	5.07 (4.29–5.85)	5.83 (5.23–6.43)	3.68 (3.00–4.36)***	−1.41 (−2.49 to −0.34)	0.01
Lysine (g)	4.83 (4.13–5.53)	4.33 (3.61–5.05)	4.90 (4.33–5.46)	2.50 (2.00–3.01)***	−1.90 (−2.89 to −0.91)	<0.001
Methionine (g)	1.62 (1.39–1.86)	1.44 (1.20–1.67)	1.66 (1.47–1.85)	0.75 (0.62–0.88)***	−0.72 (−1.05 to −0.39)	<0.001
Cysteine (g)	1.03 (0.89–1.18)	0.90 (0.76–1.03)	1.04 (0.94–1.15)	0.78 (0.65–0.91)***	−0.13 (−0.31 to +0.06)	0.19
Phenylalanine (g)	3.32 (2.89–3.75)	2.91 (2.51–3.30)	3.38 (3.03–3.72)	2.38 (1.95-2.81)***	−0.58 (−1.20 to +0.03)	0.06
Tyrosine (g)	2.50 (2.16–2.84)	2.20 (1.86–2.54)	2.58 (2.31–2.86)	1.42 (1.19–1.66)***	−0.86 (−1.34 to −0.39)	<0.001
Valine (g)	3.77 (3.24–4.29)	3.30 (2.83–3.78)	3.84 (3.44–4.24)	2.43 (2.00–2.86)***	−0.95 (−1.65 to −0.24)	0.009
Arginine (g)	4.37 (3.76–4.99)	3.91 (3.40–4.43)	4.30 (3.84–4.77)	2.98 (2.51–3.45)***	−0.87 (−1.64 to −0.09)	0.03
Histidine (g)	1.98 (1.72–2.24)	1.74 (1.48–2.00)	2.02 (1.80–2.24)	1.23 (1.01–1.44)***	−0.55 (−0.93 to −0.17)	0.005
Alanine (g)	3.56 (3.07–4.04)	3.18 (2.72–3.64)	3.60 (3.21–3.99)	2.21 (1.84–2.58)***	−1.01 (−1.67 to −0.35)	0.003
Aspartic acid (g)	6.88 (5.94–7.82)	6.21 (5.41–7.01)	6.81 (6.14–7.49)	4.91 (4.04–5.79)***	−1.23 (−2.47 to +0.01)	0.05
Glutamic acid (g)	14.22 (12.40–16.05)	12.44 (10.74–14.15)*	14.41 (12.95–15.88)	10.73 (9.01–12.45)***	−1.91 (−4.36 to +0.55)	0.13
Glycine (g)	3.11 (2.69–3.53)	2.80 (2.42–3.19)	3.14 (2.81–3.47)	1.98 (1.65–2.31)***	−0.86 (−1.43 to −0.28)	0.004
Proline (g)	4.57 (3.94–5.20)	3.86 (3.23–4.49)*	4.66 (4.19–5.13)	3.15 (2.58–3.73)***	−0.80 (−1.67 to +0.07)	0.072
Serine (g)	3.41 (2.95–3.87)	2.98 (2.56–3.40)*	3.45 (3.10–3.79)	2.44 (1.99–2.89)***	−0.57 (−1.18 to +0.03)	0.06

Data are means  ±  SD

Listed *p*-values are for interactions between group and time assessed by repeated measures ANOVA. *p <  0.05; **p < 0.01; ***p < 0.001 for within-group changes from baseline assessed by paired comparison *t*-tests

### Body composition and insulin resistance

**Fig. 2:**
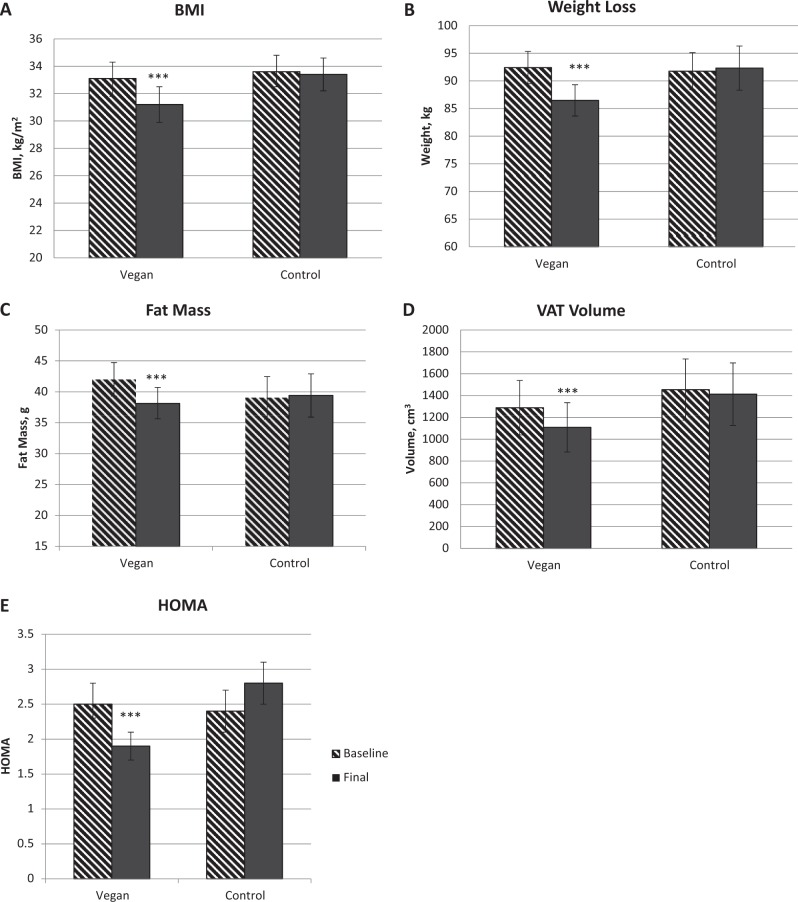
Changes in body weight, fat mass, and insulin resistance in the vegan and control group at baseline and after 16 weeks. (A) Body Mass Index (BMI), Gxt p < 0.001; (B) Body Weight, Gxt p < 0.001; ; (C) Fat Mass, Gxt p < 0.001; (D) Visceral Fat Volume, Gxt p < 0.001; and (E) Homeostatic Model Assessment Insulin Resistance (HOMA-IR), Gxt p=0.004. Gxt is interaction between group and time from the ANOVA model. *** for p < 0.001. Data are given as means with 95% confidence intervals

Significant reductions in body mass index and body weight were only observed in the vegan group (treatment effect, −2.0; 95% CI −2.6 to −1.5 kg/m^2^; Gxt, *p* < 0.001; Fig. 2a; and −6.5; 95% CI −8.9 to −4.1 kg; Gxt, *p* < 0.001; Fig. 2b, respectively). Similarly, fat mass and particularly visceral fat volume were reduced only in the vegan group (treatment effect −4.3; 95% CI −5.4 to −3.2 kg; Gxt, *p* < 0.001; Fig. 2c; and −224; 95% CI −328 to −120 cm^3^; Gxt, *p* < 0.001; Fig. 2d, respectively). Only the vegan group had significantly reduced HOMA-IR as well (treatment effect −1.0; 95% CI −1.2 to −0.8; Gxt, *p* = 0.004; Fig. 2e).

### Association between protein intake and body composition

**Fig. 3:**
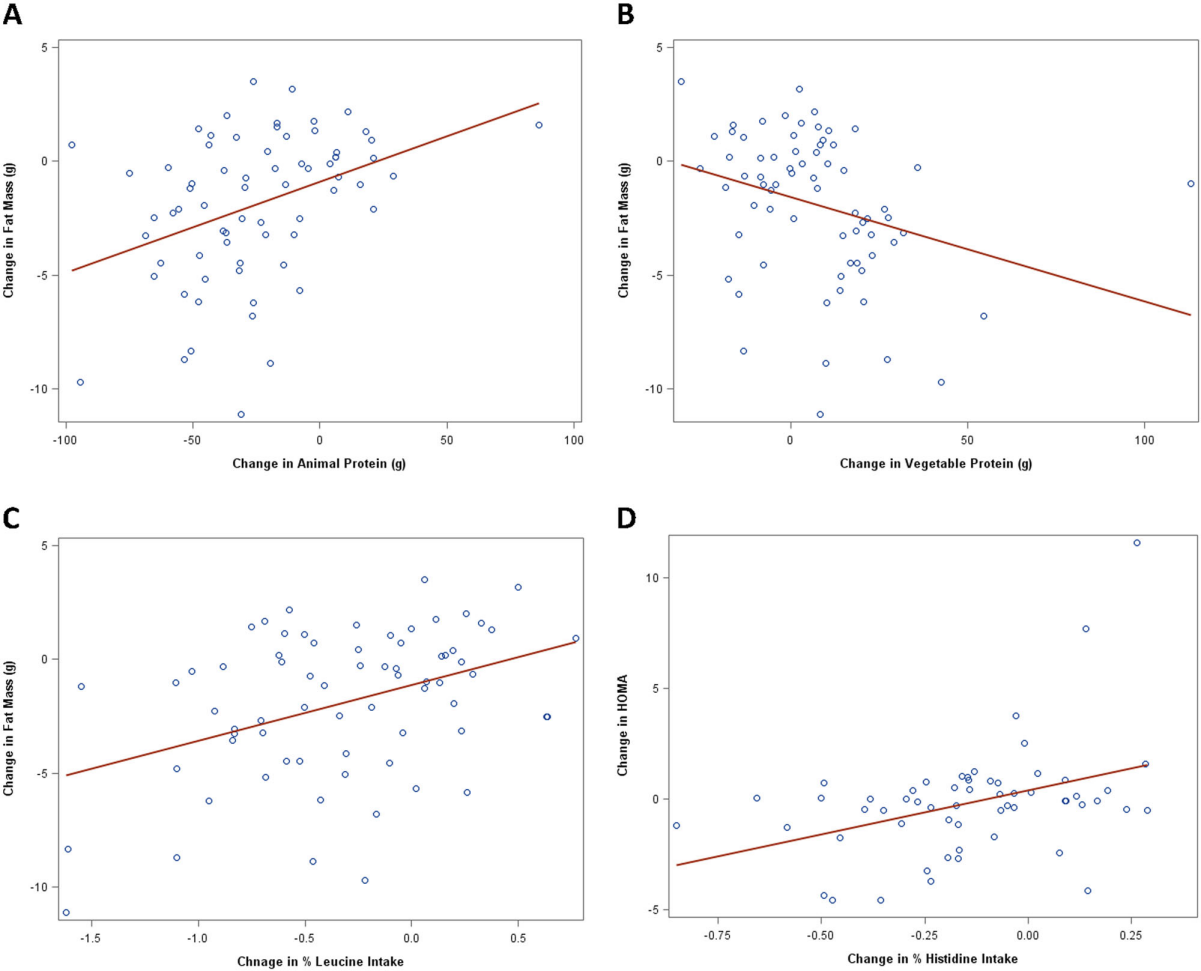
Regression models for changes in (A) Animal protein and fat mass: r = +0.39; p = 0.001; (B) Vegetable protein and fat mass: r = -0.30; p = 0.011; (C) % Leucine intake and fat mass: r = +0.40; p < 0.001; and (D) % Histidine intake and HOMA-IR (Homeostasis Model Assessment) insulin resistance: r = +0.38; p = 0.003

A decreased intake of animal protein was associated with a decrease in fat mass (*r* = +0.39; *p* = 0.001). Every 1 gram reduction in animal protein intake was associated with a reduction of 0.040 kg in fat mass. Overall, the average reduction of 36.2 g of animal protein consumption in the vegan group was associated with a reduction in fat mass of 1.45 kg (*p* = 0.001; Fig. 3a). In contrast, an increased intake of plant protein was associated with a decreased fat mass (*r* = −0.30; *p* = 0.01). Every 1gram increase in plant protein was associated with a reduction in fat mass of 0.046 kg. Overall, the average increase of plant protein of 19.2 g in the vegan group was associated with a reduction in fat mass by 0.88 kg (*p* = 0.01; Fig. 3b).

We observed a positive correlation between changes in leucine consumption as a percent of total protein and changes in fat mass. Lowering % leucine intake was associated with a decrease in fat mass (*r* = +0.40; *p* < 0.001). This was shown in the vegan group in which a 0.3% reduction in leucine intake was associated with a 0.82 kg (*p* = 0.001; Fig. 3c) reduction in fat mass, even after adjusting for changes in BMI and energy intake (*r* = +0.28; *p* = 0.033).

### Association between protein intake and insulin resistance

Lowering % histidine intake was associated with a decrease in HOMA (*r* = +0.38; *p* = 0.003). A 0.2% reduction in histidine intake in the vegan group was associated with a reduction in HOMA-IR by 0.79 (*p* = 0.003) (Fig. 3d). This association remained significant even after adjustment for changes in BMI and energy intake (*r* = +0.34; *p* = 0.01). In addition, changes in % intake of the following amino acids were also positively associated with changes in HOMA: threonine (*r* =+0.33; *p* = 0.011), leucine (*r* = + 0.31; *p* = 0.017), lysine (*r* = +0.31; *p* = 0.016), methionine (*r* = +0.32; *p* = 0.016), and tyrosine (*r* = +0.33; *p* = 0.013). These correlations were no longer significant after adjustment for changes in BMI and energy intake.

## Discussion

### Main findings

This study demonstrated that the quality and quantity of dietary protein from a plant-based vegan diet are associated with improvements in body composition, body weight, and insulin resistance in overweight individuals. A decreased intake of animal protein and an increased intake of plant protein were associated with a decrease in fat mass, by 1.45 and 0.88 kg respectively. Exchanging plant protein for animal protein explains more than half of the reduction in fat mass in the vegan group (2.33 out of 4.3 kg). A large portion of fat mass reduction may be explained by the amino acid composition of plant protein, specifically by decreased leucine intake, which was associated with a decrease in fat mass by 0.82 kg, independent of changes in BMI and energy intake. Additionally, decreased histidine intake was associated with a decrease in insulin resistance, also independent of changes in BMI and energy intake. Finally, decreased intakes of threonine, leucine, lysine, methionine, and tyrosine were each associated with a decrease in insulin resistance. However, these associations were mainly driven by weight loss.

### Plant vs. animal protein in weight regulation, body composition, and insulin resistance

Multiple randomized controlled studies have established the effectiveness of plant-based diets for weight loss^25,26^. Plant-based diets have also been shown to decrease the risk of developing diabetes in additional prospective studies^27^. The specific role of plant protein in weight regulation and metabolic health is of particular interest. In a study focusing specifically on the association between protein sources and body weight regulation using data from the European Prospective Investigation into Cancer and Nutrition study, increases in body weight were positively correlated with an increased intake of animal protein, especially in women^28^. Similarly, in a 2011 observational study, increases in animal protein consumption were found to be positively correlated with increases in BMI, while increases in plant protein intake were negatively associated with changes in BMI^29^.

Dietary protein triggers release of both insulin and glucagon^12^. Specifically, a higher intake of essential amino acids can stimulate secretion of insulin and up-regulate insulin like growth factor 1 (IGF-1)^12^. Essential amino acids are found in greater abundance in animal protein, compared to plant protein. In contrast, a higher intake of non-essential amino acids is associated with down-regulation of insulin secretion and increased glucagon secretion, resulting in stimulation of gluconeogenesis, hepatic lipid oxidation, lipolysis and reduction in both IGF-1 and cholesterol synthesis. Hepatic lipid oxidation promotes appetite control and lowers the respiratory quotient, which may play a role in body weight reduction, and may further be supported by the thermogenic effect of glucagon. Human adipocyte express IGF-1 receptors, thus down-regulation of IGF-1 activity can also promote leanness^12^. Non-essential amino acids in plant protein promote higher net glucagon activity than an omnivorous diet, promoting weight loss and reduction of LDL-cholesterol^12^.

### The role of specific amino acids in insulin resistance and weight regulation

A 2018 prospective study that included more than 1,200 adults, who were followed-up for a mean of 2.3 years, showed that higher intake of branched chain amino acids (BCAA), especially leucine, can increase insulin resistance. Participants in the highest tertile for leucine intake had a 75% higher risk of developing insulin resistance compared with people in the lowest tertile (OR 1.75; 95% CI 1.09–2.82)^16^.

Increased serum concentrations of BCAA have been associated with increased risk of type 2 diabetes and underlying metabolic abnormalities^30,31^. High serum BCAA levels activate the mammalian target of rapamycin complex 1 (mTORC1) signaling pathway, leading to inhibition of glucose transport in muscle and fat tissues^16^. Animal protein from meat and dairy products contains a high percent of leucine. Therefore, these foods may stimulate the mTORC1 pathway, thus contributing to insulin resistance, and obesity^32^.

Randomized controlled trials have shown that reduced dietary intake of BCAA promote weight loss, reduce adiposity, and improve glycemic control and metabolic health^33,34^. In our study, the vegan group consumed less than 75% of the control group’s daily grams per day of BCAA. Our data also show that reduced dietary intake of leucine, in particular, was associated with decreased fat mass and reduced insulin resistance.

Additionally, our results suggest that a decreased intake of histidine, leucine, threonine, lysine, methionine, and tyrosine were all associated with a decrease in HOMA, with histidine being the only one having a significant association independent on changes in BMI and energy intake. The vegan group reduced both its absolute and relative intake of all six of these amino acids. The significant decrease in the consumption of sulfur-containing amino acids, i.e. cysteine and methionine, in the vegan group, is of particular interest. Several studies have shown that diets restricting sulfur-containing amino acids have shown beneficial effects in the prevention of chronic diseases, including type 2 diabetes, cancer, and cardiovascular disease^14,17^. Dietary restriction of methionine and cysteine without caloric restriction has been associated with reductions in body weight, adiposity, blood levels of insulin, IGF-1, and glucose^17^, as well as reductions in cardiovascular risk factors including blood lipids, homocysteine, and blood pressure^18,19^. Our results suggest that reduced intake of methionine through a plant-based diet may correlate with a decrease in both body weight and insulin resistance.

### Meeting and exceeding the recommended daily intake on a plant-based diet

Higher animal protein consumption has been associated with increased risk of metabolic disease and mortality. A 2015 study using data from NHANES II reported the link between protein intake and mortality in men and women. Subjects in the high-protein group (consuming 20% or more of daily calories as protein) had a 73-fold increase in risk of diabetes mortality and a 74% increase in relative risk of all-cause mortality^20^. Our data suggest that both the decreased intake of animal protein and the amino acid composition of the plant-based diet are associated with decreased body fat and reduced insulin resistance.

The United States Department of Agriculture recommends a minimum of 46 g of protein per day for women and 56 g per day for men^35^. In the current study, all participants in the vegan group exceeded the recommended daily intake of protein and of each individual amino acid. While animal protein is higher in essential amino acids, containing significant amounts of leucine, histidine, threonine, methionine and lysine, consumption of plant protein, which is higher in non-essential amino acids, offers clear metabolic benefits. People following a plant based diet still consume more than 100% of the recommended dietary intake of essential amino acids. The main plant sources of these amino acids are legumes, grains, and vegetables. For example, 2 servings of oatmeal made from 100 g of oats contain 102% of recommended daily intake of tyrosine^36^.

### Strengths and limitations

Utilizing a randomized control trial allowed us to analyze the relationship between dietary protein and specific amino acids with changes in body weight and insulin resistance. This 16 week study provided an ample amount of time for participants to adapt to the diet. The low attrition rate suggests that a plant-based diet is sustainable and can be incorporated into diverse lifestyles. A limitation to this study that must be considered is the dietary assessment method. Three day dietary records at baseline and week 16 were used which likely have some degree of error due to inaccurate and therefore misrepresentative reporting. To minimize this error, participants were taught how to give detailed reports. Random periodic phone calls were also used to evaluate and monitor participants’ food records. This study could not definitively prove a causal relationship between protein intake and metabolic outcomes. Such a conclusion would require a specifically-designed randomized clinical trial. However, our study suggests overall benefits of a plant-based diet.

## Conclusions

The quantity and quality of dietary protein, as part of a plant-based diet, are associated with improvements in body weight, body composition, and insulin resistance in overweight individuals. A greater consumption of plant protein, in replacement of animal protein, resulted in decreased fat mass. More specifically, decreased leucine intake was associated with a decrease in fat mass, independent of changes in BMI. In addition, decreased intakes of histidine, threonine, leucine, lysine, methionine, and tyrosine were each associated with a decrease in insulin resistance. For histidine, this association remained significant after adjustment for changes in BMI and energy intake. Our study highlights the need for additional research to explore the mechanisms explaining the beneficial role of plant protein and specific amino acids in regulating body weight, body composition, and insulin resistance.

## Electronic supplementary material

Supplemental Figure 1
