# Supplementary material for: A plant-based diet in overweight individuals in a 16-week randomized clinical trial: metabolic benefits of plant protein
Source: Nutr Diabetes. 2018 Nov 2;8:58. doi: 10.1038/s41387-018-0067-4 (PMC6221888; doi:10.1038/s41387-018-0067-4)
Supplement: Supplementary file 1 — Supplemental Figure 1 [file 41387_2018_67_MOESM1_ESM.ppt]

## Slide 1
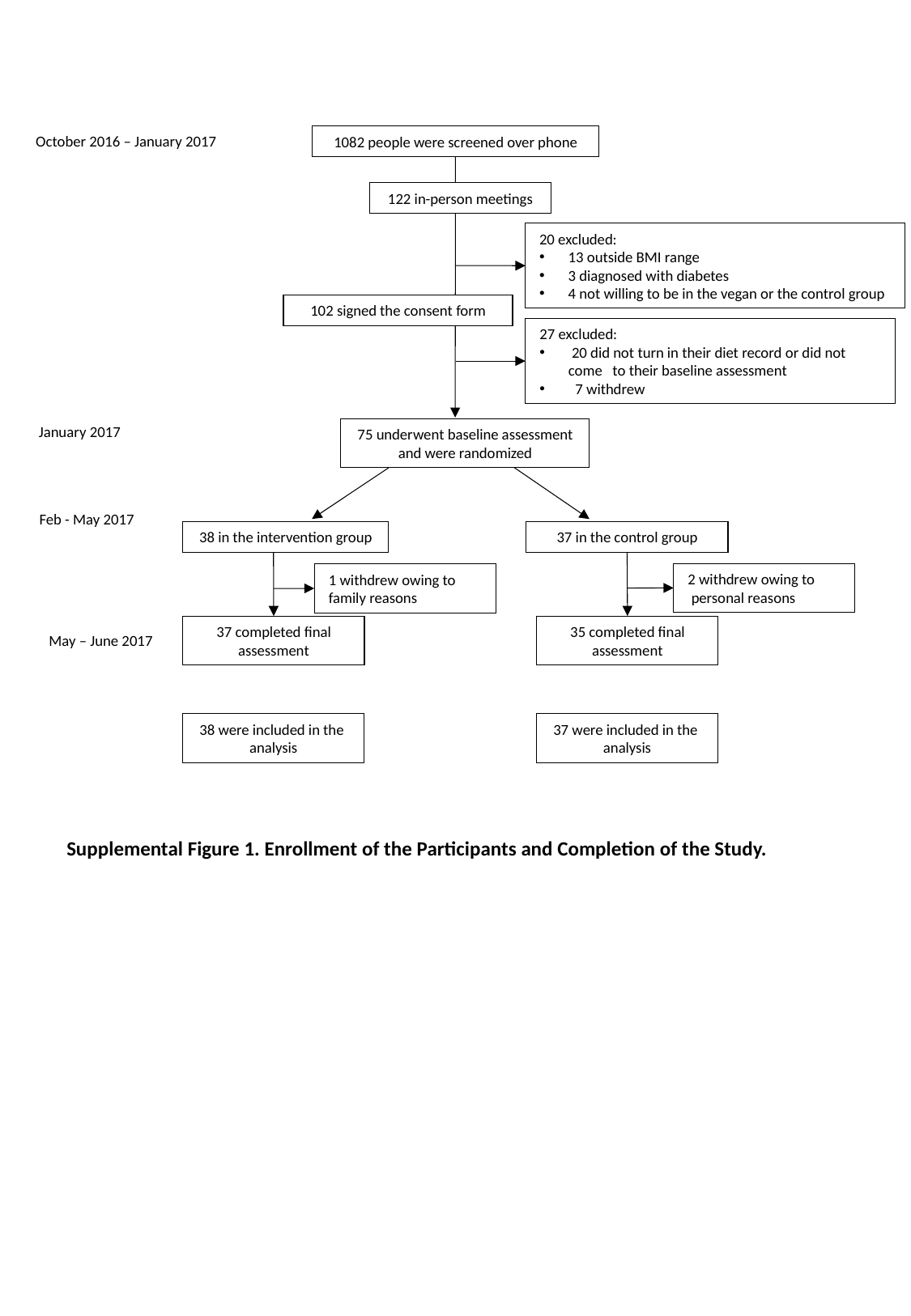

October 2016 – January 2017
1082 people were screened over phone
January 2017
38 in the intervention group
37 in the control group
2 withdrew owing to
 personal reasons
1 withdrew owing to family reasons
35 completed final assessment
37 completed final assessment
May – June 2017
38 were included in the analysis
37 were included in the analysis
122 in-person meetings
20 excluded:
13 outside BMI range
3 diagnosed with diabetes
4 not willing to be in the vegan or the control group
102 signed the consent form
27 excluded:
 20 did not turn in their diet record or did not come to their baseline assessment
 7 withdrew
75 underwent baseline assessment and were randomized
 Feb - May 2017
Supplemental Figure 1. Enrollment of the Participants and Completion of the Study.
